# Methodological Strategies for Ecological Momentary Assessment to Evaluate Mood and Stress in Adult Patients Using Mobile Phones: Systematic Review

**DOI:** 10.2196/11215

**Published:** 2019-04-01

**Authors:** Yong Sook Yang, Gi Wook Ryu, Mona Choi

**Affiliations:** 1 Mo-Im Kim Nursing Research Institute College of Nursing Yonsei University Seoul Republic of Korea

**Keywords:** review, experience sampling method, ecological momentary assessment, mobile apps, mood, stress

## Abstract

**Background:**

Ecological momentary assessment (EMA) has utility for measuring psychological properties in daily life. EMA has also allowed researchers to collect data on diverse experiences and symptoms from various subjects.

**Objective:**

The aim of this study was to review methodological strategies and useful related information for EMA using mobile phones to capture changes of mood and stress in adult patients seeking health care.

**Methods:**

We searched PubMed, Cumulative Index to Nursing and Allied Health Literature (CINAHL), Embase, the Cochrane Library, PsycINFO, and Web of Science. This review included studies published in peer-reviewed journals in English between January 2008 and November 2017 that used basic- or advanced-feature mobile phones to measure momentary mood or stress in adult patients seeking health care in outpatient departments. We excluded studies of smoking and substance addictions and studies of mental disorder patients who had been diagnosed by physicians.

**Results:**

We reviewed 12 selected articles that used EMA via mobile phones to measure momentary mood and stress and other related variables from various patients with chronic fatigue syndrome, breast cancer, migraine, HIV, tinnitus, temporomandibular disorder, end-stage kidney disease, and traumatic brain injury. Most of the selected studies (11/12, 92%) used signal contingency and in 8 of the 12 studies (67%) alarms were sent at random or semirandom intervals to prompt the momentary measurement. Out of 12 studies, 7 (58%) used specific apps directly installed on mobile phones, 3 (25%) used mobile phones to link to Web-based survey programs, and 2 (17%) used an interactive voice-response system.

**Conclusions:**

This study provides researchers with useful information regarding methodological details for utilizing EMA to measure mood and stress in adult patients. This review shows that EMA methods could be effective and reasonable for measuring momentary mood and stress, given that basic- and advanced-feature mobile phones are ubiquitous, familiar, and easy to approach. Therefore, researchers could adopt and utilize EMA methods using mobile phones to measure psychological health outcomes, such as mood and stress, in adult patients.

## Introduction

Momentary assessment techniques, such as ecological momentary assessment (EMA), have a long tradition as a prospective and repeated-measures longitudinal research methodology [1]. Originally, paper diaries were used in combination with pagers or electronic wristwatches. As technology became more advanced, data collection logistics and reliability were improved by the use of personal digital assistants and mobile phone apps [2]. The method focuses on symptoms and adaptive function, such as well-being, and aims to map daily psychological function [3]. This method captures fluctuations by taking measurements multiple times day-to-day, unlike retrospective reporting, and has produced many findings with respect to psychological properties in the daily life of subjects [4-7].

EMA methods have ecological validity because assessments are made in natural and real-life environments, which reduces recall bias and avoids aggregation since it assesses the actual moment of interest repeatedly at multiple time points [3,8]. These repeated measures over time can reduce assessment error and improve the validity, reliability, and transparency of individual pattern assessments [3]. These aspects of increasing accuracy [8] and sensitivity to changes [9] in various properties have made EMA advantageous to study psychological state, quality of life, mobility, social networks, and more [3]. This method is considered suitable for understanding daily changes in psychological features such as mood and stress [10-12]. Traditionally, mood and stress have been assessed using retrospective measures [13]. EMA methods might provide health care providers with more accurate data than retrospective and global self-reporting methods. This may increase access to effective treatments by enabling enhanced understanding of the daily mood and stress of subjects, which are closely related to environmental factors.

The prevalence of mobile phones is increasing. In addition, advanced mobile technology has rendered mobile phones a novel, plausible way to implement EMA methods utilizing mobile technology, which is already available and familiar to many populations [14-16]. In an EMA study of police officers using a mobile phone app, participants indicated that the EMA correctly measured their mood and stress; they also felt comfortable using the app installed on their own mobile phones [12].

There have been systematic reviews of EMA methods monitoring adult patients with psychiatric disorders. A review study of depressive symptoms or affective disorders showed that the monitoring system using a mobile phone-based EMA method was feasible and accurate in predicting mood, but this study did not include postpartum, postnatal, or pregnant women with depressive symptoms [17]. Another review of studies on anxiety disorders, such as panic disorder, generalized anxiety disorder, social phobia, posttraumatic stress disorder, and obsessive-compulsive disorder [18], found that EMA methods have the potential to illuminate patients’ anxiety in their everyday lives.

However, there is no extant review of the feasibility and use of EMA methodology using basic- or advanced-feature mobile phones to capture changes of mood and stress in adult patients without diagnoses of psychiatric disorders such as affective, anxiety, or mood disorders. Therefore, this review provides methodological details for the use of EMA technology to assess mood and stress in adult patients.

## Methods

### Information Source and Search Strategy

The search included studies that used mobile apps to measure momentary mood or stress in adults; the studies were published in peer-reviewed journals in English between January 2008 and November 2017. We performed database searches on six online biomedical databases—PubMed, Cumulative Index to Nursing and Allied Health Literature (CINAHL), Embase, the Cochrane Library, PsycINFO, and Web of Science. We also performed hand-searches of the Journal of Medical Internet Research (JMIR) and the website of the Society for Ambulatory Assessment. We used the following search terms: (“ecological momentary assessment” [MeSH] OR “experience sampling” OR “ecological momentary” OR “event sampling” OR “ambulatory assessment” OR “structured diary method” OR “real-time data capture studies” OR “real-time data capture study” OR “beeper studies” OR “beeper study” OR “intensive longitudinal assessment”) AND (“stress, psychological” [MeSH] OR “affect” [MeSH] OR “mood” OR “emotion” OR “affection” OR “stress”) AND (“mobile applications” [MeSH] OR “smartphone” [MeSH] OR “cell phones” [MeSH] OR “smartphone*” OR “cell phone” OR “cellular phone” OR “mobile app*”). The articles identified were inspected, including their reference lists and in-text citations of relevant articles (see Multimedia Appendix 1).

### Study Selection

Studies were included that used basic- and advanced-feature mobile phones to measure momentary mood or stress in adult patients. We included those studies that were published in peer-reviewed journals in English. Specifically, included studies used basic- or advanced-feature mobile phones to deliver EMAs. Included studies also involved adult patients in community settings who were diagnosed with a certain disease by their physicians and cared for in outpatient settings. We also included studies that involved people who had mood or stress problems without diagnosis by their physicians of psychiatric disorders, such as affective, anxiety, and mood disorders or of substance addictions. The year 2008 was chosen as the earliest year of publication because the first app downloaded on a mobile device was in 2008 [19]. Studies were excluded if they were studies of smoking, diet, addictions, major psychological problems, or child populations.

### Screening Procedure

**Figure 1:**
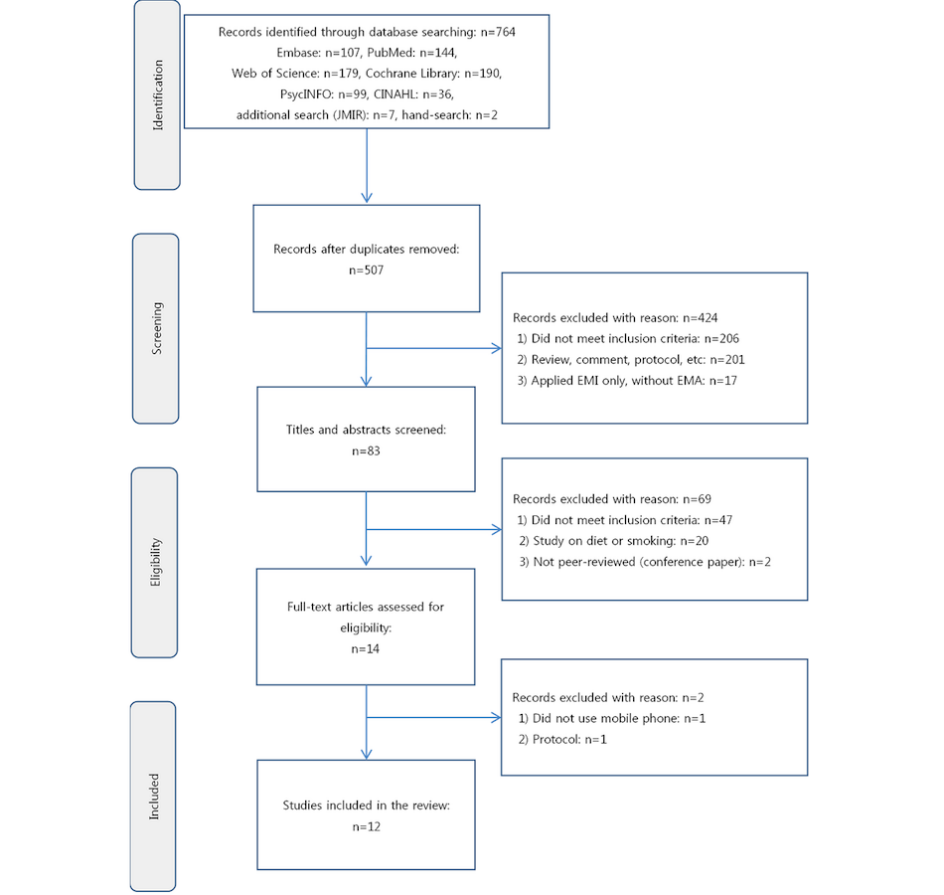
Preferred Reporting Items for Systematic Reviews and Meta-Analyses (PRISMA) flowchart providing an overview of the study selection process. CINAHL: Cumulative Index to Nursing and Allied Health Literature; EMA: ecological momentary assessment; EMI: ecological momentary intervention; JMIR: Journal of Medical Internet Research.

A total of 764 articles were retrieved from the six databases, in which 257 records were duplicated. For 507 articles, two reviewers (YSY and GWR) independently screened titles and abstracts. After that, the same two reviewers independently reviewed full-text articles to decide whether each article was relevant to the review. In case of disagreement, a third person (MC) was consulted to reach consensus. Ultimately, 12 full-text articles were selected according to the criteria and relevant data were extracted. Figure 1 shows the process of study selection based on the Preferred Reporting Items for Systematic Reviews and Meta-Analyses (PRISMA) guidelines [20].

### Data Extraction

The following information was extracted: study purpose, sample characteristics, main momentary measurement, data analysis method, and methodological details of EMA, such as operating system, mode of response, contingency, duration of data collection, frequency, and alarm interval for each study.

## Results

In total, 12 studies met the selection criteria. The following sections summarize how EMA approaches were applied to the study populations along with methodological details.

### Subject Characteristics and Main Momentary Measurements

Various clinical populations were included in this review: patients with chronic fatigue syndrome [21,22], HIV [5,23-25], tinnitus [6], migraine [26], minor traumatic brain injury [27], breast cancer [7], end-stage kidney disease [28], and temporomandibular disorder [29]. Out of 12 studies, 11 (92%) [5,7,21-29] measured mood or stress as major variables by the EMA method; 1 study (8%) assessed stress or stressors [6]; and 5 studies (42%) [5,6,24-26] measured mood or affect along with stress or stressors. A total of 2 studies out of 12 (17%) measured pain along with mood or stress [26,29]. The majority of articles (11/12, 92%) captured changes in mood or stress over time without applying interventions [5-7,21-28]; 1 article out of 12 (8%) reported changes of mood from a pre-post approach [29] (see Table 1).

**Table 1:** Study details including study purpose, sample characteristics, and main momentary measurements.

Author (year), country	Study purpose	Sample characteristics	Sample size, n	Age in years	Main momentary measurements
Band et al (2016) [21], United Kingdom	To examine relationship between significant others’ responses and patient outcomes	Pairs of CFS^a^ patients and significant others	23	35.5 (14.0)^b^	Affects, significant others’ responses, symptom severity, disability, and activity management strategies
Band et al (2017) [22], United Kingdom	To investigate whether activity patterns occurred according to patient symptom experience and affect	CFS patients	23	35.5 (14.0)^b^	Patient activity management strategies, patient affects, and symptoms
Farmer et al (2017) [5], United States	To assess stress, frequency of stressors, stressful life events, and behaviors	Patients with HIV	32	46.0 (23-64)^c^	Stressors, stress level, emotional and physical states, medication adherence, and sexual activity
Moore et al (2017) [23], United States	To examine feasibility, acceptability, and initial validity of using mobile phone-based EMA^d^	Older adults with HIV	20	58.8 (4.3)^b^	Mood and cognitive symptoms
Cook et al (2017) [24], United States	To test whether momentary motivation was a mechanism by which everyday experiences affect medication adherence	Patients with HIV	87	40.0 (8.8)^b^	Control beliefs, mood, stress, coping, and social support
Cook et al (2017) [25], United States	To test predictors of electronically monitored adherence at both the state and trait levels and to compare relative effects	Patients with HIV	87	40.0 (8.8)^b^	Thoughts, mood, stress, coping, social support, and treatment motivation
Wilson et al (2015) [6], United States	To explore feasibility of EMA as a tool to more accurately assess the level of bother from tinnitus	Tinnitus patients	20	55 (38-65)^c^	Bother, loudness, and stress
Houtveen et al (2013) [26], the Netherlands	To test prodromal functioning relative to the interictal state	Migraine patients	87	44.5 (25-68)^c^	Migraine attacks and prodromal features: fatigue, cognitive functioning, affects, and stressors
Juengst et al (2015) [27], United States	To assess pilot feasibility and validity of a mobile health system for tracking mood-related symptoms after traumatic brain injury	Traumatic brain injury patients	20	36.7 (12.4)^b^	Depressive and anxious mood, impact of fatigue, and affects
Kim et al (2016) [7], South Korea	To evaluate the potential of a mobile mental health tracker, the impact of adherence on reporting, and its accuracy	Breast cancer patients	78	44.4 (7.0)^b^	Sleep satisfaction, mood, and anxiety
Abdel-Kader et al (2014) [28], United States	To evaluate day-to-day and diurnal variability of fatigue, sleepiness, exhaustion, and related symptoms	End-stage kidney disease patients	55	56.7 (17.3)^b^	Mood, cognition, sleepiness, and exhaustion
Litt et al (2009) [29], United States	To determine whether cognitive-behavioral therapy treatment operates by effecting changes in cognitions, affects, and coping behaviors	Temporomandibular disorder patients	54	41.0 (11.9)^b^	Pain, coping, and affects

^a^CFS: chronic fatigue syndrome.

^b^Mean (SD).

^c^Median (range).

^d^EMA: ecological momentary assessment.

Out of 12 studies, 2 studies of patients with chronic fatigue syndrome (CFS) (17%) assessed patients’ affect [21,22]. Out of these 2 studies, 1 (50%) focused on activity management strategies, affect, and symptoms to investigate whether activity patterns occurred according to patients’ symptom experience and affect [22]. The other study (1/2, 50%) examined the relationship between significant others’ responses and patient outcomes such as affect, symptom severity, disability, and activity management strategies [21].

Out of 12 studies, 4 EMA studies of HIV patients (33%) measured several variables. Out of these 4 studies, 1 (25%) evaluated momentary mood and cognitive symptoms of HIV patients [23], and another (1/4, 25%) assessed control beliefs, mood, stress, coping, and social support to examine whether momentary motivation is a mechanism by which everyday experiences affect adherence to medication therapy [24]. Cook et al’s study (1/4, 25%) measured thoughts, mood, stress, coping, social support, and treatment motivation to test predictors of electronically monitored adherence at both state and trait levels [25]. Out 4 studies, 1 EMA study (1/4, 25%) also investigated stress, frequency of stressors, stressful life events, and behaviors of HIV patients [5].

Out of 12 studies, 1 (8%) assessed experience of migraine attacks and prodromal features, such as fatigue, cognitive functioning, affect, effort spent (eg, working hard and feeling strained), and stressors, to test and identify individual prodromal features related to the interictal state in moderate-to-severe migraine patients [26].

In a study of minor traumatic brain injury patients (1/12, 8%), mood and affect were assessed to evaluate feasibility and validity of a mobile health system app [27]. A study of patients with breast cancer (1/12, 8%) measured sleep satisfaction, mood, and anxiety to evaluate the potential of a mobile, mental health tracker app using daily mental health ratings as indicators of depression [7].

Out of 12 studies, 1 (8%) evaluated day-to-day and diurnal variability of fatigue, sleepiness, exhaustion, and related symptoms in end-stage kidney disease patients [28]. Out of 12 studies, a pre-post EMA design in 1 study (8%) was applied to measure pain, coping, and affect in order to evaluate the effect of cognitive-behavioral treatment for temporomandibular disorder patients in the context of painful episodes [29].

In terms of main momentary measurement, half of the included studies (6/12, 50%) measured momentary mood, affect, or stress with standardized scales for validation [7,23-25,27,29], while others (6/12, 50%) did not administer or specify them [5,6,21,22,26,28]. Band et al’s study (1/12, 8%) captured mood changes by using two subscales of positive affect and negative affect of CFS patients [22]. In this study, positive affect was assessed using five items: excited, happy, satisfied, relaxed, and cheerful (Cronbach alpha=.87); negative affect was assessed using five items: sad, annoyed, irritated, anxious, lonely, and guilty (Cronbach alpha=.87). In the other study of CFS patients (1/12, 8%), affect was measured by a single item, *feeling distressed*, which was included with standard items examining patients’ affect at a momentary level [21].

The standardized measures of the Beck Depression Inventory-II and the Profile of Mood States were administered to measure state mood and stress in comparison to the momentary item for assessing mood of older adults with HIV; correlates with state mood (ie, sadness, happiness, and tiredness) and stress were evaluated by item questions developed in the study [23].

In a study of HIV patients (1/12, 8%) [24], three items for mood (Cronbach alpha=.93) and six items for stress (Cronbach alpha=.67) from the Diary of Ambulatory Behavioral States were used after piloting [30]. Another study of HIV patients (1/12, 8%) used the mood scale from the Diary of Ambulatory Behavioral States and the stress scale from the Daily Hassles Scale; they were validated by the trait measurement tools from the Center for Epidemiological Studies-Depression scale and the HIV/AIDS-Targeted Quality of Life instrument [25]. Both trait-level mood and stress predicted their respective state-level measures.

In 1 study out of 12 (8%), the Daily Mood and Affect scale for momentary assessment was developed; the Positive and Negative Affect Schedule and the 9-item Patient Health Questionnaire as standardized measures were applied [27]. In a study of breast cancer patients (1/12, 8%), the author used 3-item short scales for anxiety, mood, and sleep satisfaction, rated by facial emoticon scales, and evaluated the concurrent validity with the standardized mood scale of the 9-item Patient Health Questionnaire [7].

In 1 study out of 12 (8%), evaluating the effects of cognitive-behavioral therapy of patients with temporomandibular pain, a standardized tool—the Center for Epidemiological Studies-Depression scale—was used to compare pre- to posttreatment change of affect using a mood item borrowed from the Coping Strategies Questionnaire [29].

Out of 12 studies, 4 (33%) reported on feasibility or validity of an EMA app [6,23,26,27]. A study with EMA design for patients suffering from tinnitus (1/4, 25%) indicated that they would suggest an EMA method to a friend [6]. Participants expressed their experience with the EMA method positively [23,27]; they reported that they accepted it as usable and were satisfied with the EMA method [26,27].

An evaluation of the usefulness or perceptions by participants of the EMA methods was conducted in another study (1/12, 8%); the results indicated that the EMA using mobile phones was useful and reliable for self-monitoring of functioning ability in daily routines [5]. EMA showed promising results in the field of screening depressive moods in a clinical population by evaluating accuracy of depression screening via the EMA method (1/12, 8%) [7].

### Methodological Details of Ecological Momentary Assessment

Table 2 shows information on methodological details of EMA used in the studies, such as the operating system of mobile phones, mode of response, contingency, duration of data collection, frequency per day, and alarm interval. Different operating systems were used to install the mobile apps, but more than half of the studies (7/12, 58%) used Android operating systems [5,7,21,22,24,25,27].

**Table 2:** Completion rate and momentary data analysis method.

Author (year)	Operating system	Mode	Contingency	Duration in days, n	Frequency per day, n	Total frequency, n	Alarm interval
Band et al (2016) [21]	Android	App	Signal	6	10	60	Semirandom
Band et al (2017) [22]	Android	App	Signal	6	10	60	Semirandom
Farmer et al (2017) [5]	Android	App	Signal and event	42	1 (medication adherence); 4 (emotional and physical states); 7 (stressor)	42 (medication adherence); 168 (emotional and physical states); 294 (stressor)	Fixed; fixed; self-initiated time (event-based)
Moore et al (2017) [23]	Android	App	Signal	7	5	35	Fixed (adjusted for participant)
Cook et al (2017) [24]	Android	Link to online survey	Signal	70	1	70	Random
Cook et al (2017) [25]	Android	Link to online survey	Signal	70	1	70	Random
Wilson et al (2015) [6]	Not specified	Link to online survey	Signal	14	4	56	Random (09:00-20:00)
Houtveen et al (2013) [26]	Nokia	App	Signal	28	4	112	Random (09:30-16:00); semirandom at get-up time and bedtime
Juengst et al (2015) [27]	Not specified	App	Signal	56^a^	1	56	Fixed by preference
Kim et al (2016) [7]	Not specified	App	Not specified	336	1	336	Not specified
Abdel-Kader et al (2014) [28]	Not specified	IVR^b^	Signal (call)	7	4	28	Fixed
Litt et al (2009) [29]	Not specified	IVR	Signal (call)	7 (pre); 14 (post)	4	28; 56	Random (08:00-22:00)

^a^Repeated four times over 8 weeks.

^b^IVR: interactive voice response.

Out of the 12 studies, 7 (58%) used specific apps directly installed onto mobile phones [5,7,21-23,26,27]; 3 studies (25%) used a Web-based online survey program hyperlinked from the mobile phones [6,24,25]; and the remaining 2 studies (17%) applied an EMA method using an interactive voice-response system [28,29]. A daily repeated voice-recorded EMA design could be a good system for patients with motor dysfunction, instead of a mobile phone app or online survey in which patients have to operate the phones to respond.

Out of 12 studies, 11 (92%) [5,6,21-29] included in the review used signal contingency to prompt momentary measurement; there was 1 study (8%) where the contingency method was not specified [7]. In a study of patients with HIV (1/12, 8%), both signal-based contingency and event-based self-report were applied [5]. Frequency of the contingency varied from once per day [7] to 10 times per day [21,22], and the study durations ranged from a minimum of 6 days [21,22] to a maximum of 48 weeks, which equals 336 days [7].

The studies with the shortest period (2/12, 17%) had the highest frequency per day of assessment [21,22], and studies with lengthy periods of more than 6 weeks (4/12, 33%) had the lowest frequency [7,24,25,27]. Out of 12 studies, 1 (8%) tried various frequencies of momentary assessment by constructs: once a day for measuring medication adherence, four times a day for emotional and physical states, and seven times per day for stressors [5].

The interval of the reminder signal varied according to the study design from random, stratified semirandom, and semirandom to fixed time per participant. A total of 4 studies out of 12 (33%) had set the alarm time as fixed according to the preference or convenience of each participant to improve compliance to the EMA [5,23,27,28].

Study completion rates ranged from 64.6% [22] to 89.5% [26], excluding studies with no reported completion rates (see Table 3). A study of temporomandibular disorder patients (1/12, 8%) paid participants US $5 for every day that they completed at least 50% of scheduled daily assessments [29], while 2 studies of HIV patients (17%) provided incentives of US $25 and the mobile phone used in the study when they finished the EMA measurement [24]. In another study of patients with HIV (1/12, 8%), in which both signal-based and event-based EMA methods were applied, event-based self-reports were encouraged by applying incentives up to US $70 to reach the survey goal of seven times per day [5]. However, the study did not calculate completion rate, since the measure was reported in an event-based way [5]. Other studies included in this review (6/12, 50%) did not mention incentives [21-23,26-28]. No articles evaluated related factors affecting the completion rate.

Because EMA datasets include diverse sources of variance, various analysis methods have been employed to address the complexity and hierarchy of the data. Out of the 12 studies, 7 (58%) reviewed here undertook multilevel or mixed-modeling analysis [21,22,24-26,28,29]. A total of 2 studies out of 12 (17%) used the MTMIXED command in Stata (StataCorp LLC) for continuous outcome variables in multilevel modeling [21,22]. Linear mixed-model multilevel analysis with maximum likelihood estimation was employed [24-26] (see Table 3).

Of the 12 studies, 2 (17%) used descriptive analysis and correlation analysis [23,27], and 2 others (17%) applied the receiver operating characteristic and ordinary least squares according to the characteristics of the variables analyzed [6,7]. Kim et al’s study [7] (1/12, 8%) estimated random-effects logistic regression parameters and thereafter used receiver operating characteristic plots to evaluate the screening accuracy of the model.

Of the 12 studies, 1 study of HIV patients (8%) applied EMA using both quantitative and qualitative measurement with various frequencies according to the target variables. The data analysis method for quantitative data was not specified, while a grounded thematic coding method in Dedoose (SocioCultural Research Consultants LLC), a Web-based mixed-method data analysis program, was applied for qualitative data of the user experience of the usefulness or perceptions regarding the EMA app [5].

While there is no standard for appropriate response rate to assess validity, 1 study out of 12 (8%) clarified that they used all available daily observations [24], and another (1/12, 8%) excluded participants who completed fewer than 20 assessments out of the total of 60 for preliminary analysis but retained all participants in the final analysis [22]; other studies did not specify inclusion criteria for response rate or number of observations for statistical analysis. A study of temporomandibular pain patients (1/12, 8%) used the observations selectively, in accordance with the study purpose, in which pain was nonzero and coping was recorded at the same time [29].

Of the 12 studies, 7 (58%) had a briefing or intake session to ensure that participants understood the EMA app before starting the survey. Participants could practice and ask questions regarding the app during the session. Informed consent and non-EMA measures, such as baseline or laboratory measurement, were also obtained during the session. After finishing the EMA phase, patients were debriefed to evaluate their experiences during the study.

**Table 3:** Completion rate and method used to analyze momentary data.

Author (year)	Completion rate of EMA^a^, n/N (%) or % (where n/N was not available)	Analysis method
Band et al (2016) [21]	38.74/60 (65)	Multilevel models
Band et al (2017) [22]	893/1380 (64.71)	Multilevel models
Farmer et al (2017) [5]	Not reported	Ground thematic coding method (not specified for quantitative data analysis)
Moore et al (2017) [23]	30/35 (86)	Descriptive and correlation analysis
Cook et al (2017) [24]	73.0	Multilevel modeling analysis
Cook et al (2017) [25]	65.0	Multilevel modeling analysis
Wilson et al (2015) [6]	889/1120 (79.38)	Ordinary least squares robust regression analysis
Houtveen et al (2013) [26]	89.5	Linear mixed-model multilevel analysis
Juengst et al (2015) [27]	73.4	Descriptive and correlation analysis
Kim et al (2016) [7]	Not reported	Random-effect model of logistic regression and receiver operating characteristic
Abdel-Kader et al (2014) [28]	1252/1540 (81.30)	Linear mixed model
Litt et al (2009) [29]	72.0 (pre); 71.0 (post)	Mixed model

^a^EMA: ecological momentary assessment.

## Discussion

### Principal Findings

This review identified mobile phone-based systems for monitoring mood or stress of patients seeking health care in outpatient departments. Studies focused on EMA methods using mobile phones, which are feasible for measuring stress and mood in adult patients and elucidating relevant methodological details. The EMA methods used in the included studies were evaluated as feasible for recognizing changes with significant variation in assessment variables [27-29] and for measuring mood and stress of patients [6,7,23,27]. This review presented strategic information on EMA methods, such as mode of response, ways of sending alarm contingencies, time intervals, frequencies, and study durations, along with information about the participants in the survey and the momentary measurements.

The studies in our review used three different modes of EMA response on mobile phones: via mobile app [5,7,21-23,26,27]; via hyperlink to online survey [6,24,25]; and via interactive voice response system [28,29]. Mode of response can be selected in accordance with participants’ clinical conditions.

EMA methods are time-consuming and demanding [4]. Not all patients are willing to participate or comply strictly with the protocol. The studies included in this review showed completion rates that ranged from 64.6% to 89.5%, which was contingent on the nature of the participants. Although there is no agreed-upon gold standard for an acceptable compliance rate in EMA studies, Stone and Shiffman [31] noted that EMA data would not be representative of participants’ daily lives if compliance was lower than 80%, while another study considered that analysis using observations of participants who responded over 75% of the time would be reasonable [6].

One challenge is the complexity of EMA data [32]. An EMA protocol usually must consider item selection, period, intensity, signaling algorithm, event recording, application type, and data storage. Our review showed that the frequency of data collection varied from 1 to 10 times per day over a time period of 6 days to 48 weeks. Repeatedly answering the same questions in an EMA method requires substantial involvement, which increases the respondent’s burden, and this aspect can be frustrating for participants [33]. Related to this complexity of data collection, missing data also presents a limitation [31].

Regarding data analysis, EMA studies tend to produce multilevel datasets from multiple participants who answer a set of questions at multiple times. Therefore, standard linear and logistic regression analysis techniques are insufficient for analysis of EMA datasets. The complexity of EMA data analysis could hinder researchers and clinicians in using this method [5]. This should be taken into account when considering this technology-driven approach.

A limitation of this review is that we did not include studies that utilized other mobile devices, such as wearable sensors or personal digital assistants, since the purpose of this review was to provide insight into methodological strategies for EMA using mobile phones to assess mood or stress.

Future studies would include objective measures of related variables, such as heart rate, physical activity, and walking, which may be affected by mood and stress, to confirm dynamic relationships between symptoms and mood and stress. Additionally, multidisciplinary research involving areas such as medical diagnosis, consultation, nursing care, and ecological momentary interventions (EMIs) with EMA data collection could be an interesting focus. Through these multiple approaches, we expect to perform more accurate and valid mental and physical health monitoring and to provide optimized medical service for patients by applying patient-specific health care interventions.

### Conclusions

Prevalence of basic- and advanced-feature mobile phones is high, and mobile technology is readily used as a ubiquitous resource. Mobile phones can be utilized easily in health research to assess patients’ experiences in their daily lives, as they are convenient for patients to carry and are user friendly. In addition, patients may feel comfortable using their own familiar mobile phones with EMA methods installed.

This review provides researchers with information regarding methodological details, such as length of administration period, mode of response, contingency of sending alarms, frequencies and durations, incentives for improving compliance, and statistical methods for data analysis when utilizing EMA to measure mood and stress in adult patients.

Despite the limitations of this study, we believe this review shows that EMA is an effective and reasonable way of measuring momentary mood and stress in an era in which mobile phones are ubiquitous in the general population, including patients. In particular, individuals who have experienced mood changes or stress can benefit from EMA methods by using mobile phones to monitor or track their mood and stress vulnerabilities. This review supports the use of EMA methods to evaluate mood and stress and recommends that researchers utilize EMA methods to measure psychological health outcomes of mood and stress in various patient populations.

## Appendix

Multimedia Appendix 1 Search terms and results.

## Abbreviations

CFS: chronic fatigue syndrome
CINAHL: Cumulative Index to Nursing and Allied Health Literature
EMA: ecological momentary assessment
EMI: ecological momentary intervention
IVR: interactive voice response
JMIR: Journal of Medical Internet Research
PRISMA: Preferred Reporting Items for Systematic Reviews and Meta-Analyses
