# Supplementary material for: Methodological Strategies for Ecological Momentary Assessment to Evaluate Mood and Stress in Adult Patients Using Mobile Phones: Systematic Review
Source: JMIR Mhealth Uhealth. 2019 Apr 1;7(4):e11215. doi: 10.2196/11215 (PMC6462888; doi:10.2196/11215)
Supplement: Multimedia Appendix 1 [file mhealth_v7i4e11215_app1.pdf]

EMBASE

Search	Query	Item found
#1	ecological momentary assessment'/exp OR 'experience sampling' OR 'ecolological momentary' OR 'event sampling' OR 'ambulatory assessment' OR 'structured diary metho'd OR 'real-time data capture studies' OR 'real-time data capture study' OR 'beeper studie's OR 'beeper study' OR 'intensive longitudinal assessment'	2,604
#2	affect'/exp OR 'mental stress'/exp OR 'mood'/exp OR 'stress'/exp OR 'affection'/exp OR 'emotion'/exp'	692,888
#3	'mobile application'/exp OR 'mobile phone'/exp OR 'smartphone'/exp OR 'mobile app' OR 'cell phone' OR 'cellphone' OR 'smart phone' OR 'smartphone' OR 'cellular phone'	22,368
	#1 ANd #2 AND #3	107

PUBMED

Search	Query	Item found
#1	("Ecological Momentary Assessment"[Mesh] OR experience sampling OR ecological momentary OR event sampling OR ambulatory assessment OR structured diary method OR real-time data capture studies OR real-time data capture study OR beeper studies OR beeper study OR intensive longitudinal assessment)	29,694
#2	("Stress, Psychological"[Mesh]) OR "Affect"[Mesh]) OR (affection OR mood OR emotion OR stress)	1,524,431
#3	("Mobile Applications"[Mesh]) OR "Smartphone"[Mesh]) OR "Cell Phones"[Mesh]) OR (mobile app OR smartphone OR smart phone OR cell phone OR cellphone OR cellular phone)	18,992
	#1 AND #2 AND #3	144

Web of Science

Search	Query	Item found
#1	TS=("ecological momentary assessment" OR experience sampling OR ecological momentary OR event sampling OR ambulatory assessment OR structured diary method OR real-time data capture studies OR real-time data capture study OR beeper studies OR beeper study OR intensive longitudinal assessment)	191,595
#2	TS=("Stress, Psychological" OR "Affect" OR affection OR mood OR emotion OR stress)	2,182,200
#3	TS=("Mobile Applications" OR "Smartphone" OR "Cell Phones" OR mobile app OR smartphone OR smart phone OR c	21,023
	#1 AND #2 AND #3	179

Cochrane Library

Search	Query	Item found
#1	MeSH descriptor:[ecological momentary assessment] explode all trees OR experience sampling OR ecological momentary OR event sampling OR ambulatory assessment OR structured diary method OR real-time data capture studies OR real-time data capture study OR beeper studies OR beeper study OR intensive longitudinal assessment	7,530
#2	MeSH descriptor:[affect] explode all trees OR MeSH descriptor;[stress, psychological] explode all trees OR mood OR affection OR emotion OR stress	53,318
#3	MeSH descriptor "Mobile Applications" explode all trees OR MeSH descriptor "Cell Phones" explode all trees OR MeSH descriptor "Smartphone" explode all trees OR mobile app OR cell phone OR cellphone OR smart phone OR smartphone OR cellular phone	2,427
	#1 AND #2 AND #3	190

PsycINFO

Search	Query	Item found
#1	"ecological momentary assessment"[Mesh] OR experience sampling OR ecological momentary OR event sampling OR ambulatory assessment OR structured diary method OR real-time data capture studies OR real-time data capture study OR beeper studies OR beeper study OR intensive longitudinal assessment	14,928
#2	"Mobile Applications"[Mesh] OR "Cell Phones"[Mesh] OR "smartphone"[Mesh] OR mobile app OR cell phone OR cellphone OR smartphone OR cellular phone	6,333
#3	"Stress, psychological"[Mesh] OR "affect"[Mesh] OR affection OR mood OR stress OR emotion	464,804
	#1 ANd #2 AND #3	99

CINAHL

Search	Query	Item found
#1	Ecological Momentary Assessment[mesh] OR experience sampling OR ecological momentary OR event sampling OR ambulatory assessment OR structured diary method OR real-time data capture studies OR real-time data capture study OR beeper studies OR beeper study OR intensive longitudinal assessment	1,387
#2	Affect[mesh] OR Stress, psychological[mesh] OR mood OR affection OR Stress OR emotion	188,715
#3	Mobile Application[mesh] OR Cell Phones[mesh] OR smartphone[mesh] OR mobile app OR cell phone OR cellphone OR smartphone OR smart phone OR cellular phone	5,814
	#1 AND #2 AND #3	36

Additional search: Mhealth uhealth/ JMIR mental

Search	Query	Item found
#1	"ecological momentary assessment"[Mesh]	1,336
#2	"Mobile Applications"[Mesh] OR "Cell Phones"[Mesh] OR "smartphone"[Mesh] OR mobile app OR cell phone OR cellphone OR smartphone OR cellular phone	1,438,363
#3	"Stress, psychological"[Mesh] OR "affect"[Mesh] OR affection OR mood OR stress OR emotion	79,797
	#1 ANd #2 AND #3	7

Hand Search (The Society for Ambulatory Assessment)

Search	Query	Item found
#1	"ecological momentary assessment"	352
#2	"mobile application, cell phone, smartphone"(of 352 above)	2
